# Amino acid requirements in horses

**DOI:** 10.5713/ajas.20.0050

**Published:** 2020-03-12

**Authors:** Chan Hee Mok, Kristine L. Urschel

**Affiliations:** 1 Department of Animal and Food Sciences, University of Kentucky, Lexington, KY 40546, USA; 2 Department of Veterinary Science, Gluck Equine Research Center, University of Kentucky, Lexington, KY 40546, USA

**Keywords:** Equine, Protein Digestibility, Requirement Evaluation, Threonine, Amino Acid Nutrition

## Abstract

Evaluating amino acid requirements, specifically threonine requirements, in horses will enable better feed formulation and result in economic production, improved animal health, and reduced environmental pollution. However, the current knowledge of protein and amino acid requirements in horses is still limited. Because horses have a unique digestive system and consume a variety of feed ingredients, their protein digestibility may be affected than other species by different feed composition, and thus amino acid requirements are susceptible to vary between situations. Therefore, a careful evaluation of amino acid requirements with a proper method is needed for various conditions. This review will also provide comprehensive information that needs to be considered when designing an amino acid requirement study in horses.

## INTRODUCTION

Provision of an optimal nutrient composition in an animal’s diet is a basic and essential part of the livestock industry; therefore, a proper evaluation of nutrient requirements is fundamental. For amino acids in particular, having the requirements not only optimizes productivity, but also minimizes adverse effects caused by excessive nitrogen intake. Without accurate requirements for each indispensable amino acid, protein sources may be overfed to meet or exceed the requirements, which is not economically efficient and can cause many unfavorable results in animals, handlers, as well as the environment. Thus, evaluating requirements of individual indispensable amino acids is warranted to formulate cost-effective and well-balanced diets.

Managing body protein accretion and maintenance is crucial for the equine industry because horses are an athletic species with a high percentage of body weight (BW) as muscle. However, there is a paucity of previous research that has investigated specific requirements for crude protein (CP) and amino acids in horses at any age. Despite the fact that threonine has been suggested as the second limiting amino acid in a typical equine diet [1–3], even the NRC [4] provides only CP and lysine requirements in horses.

Conventionally, amino acid requirements in animals have been estimated based on vari ous indexes, such as amino acid composition in body tissues, growth rate, blood levels of certain metabolites related to protein metabolism, nitrogen balance in the body, or isotopic amino acid metabolism. These approaches have their own advantages and limitations. Therefore, understanding the principles of these methods as well as protein metabolism in horses is required to select an appropriate methodology for a specific situation and, consequently, to evaluate accurate amino acid requirements.

The aim of the present review is to provide comprehensive information for studying amino acid requirements in horses. The current knowledge in protein digestion and utilization in horses should be fully understood prior to designing a study for amino acid requirements evaluation. To increase the accuracy of the estimation, factors that could affect amino acid requirements, such as digestibility, endogenous losses, and various physiological states, need to be carefully considered, and a suitable method should be applied.

## CRUDE PROTEIN AND AMINO ACID REQUIREMENTS IN HORSES

The CP requirements for maintenance horses recommended in the NRC [4] are determined at the point of zero balance of nitrogen, where nitrogen accretion and losses occur at the same rate. However, measuring true nitrogen losses is susceptible to underestimation because minor sources of nitrogen losses, such as hair, skin, and sweat, are hard to quantify. Therefore, when using CP requirements evaluated by nitrogen balance trials for feed formulation, some compensation might need to be added to have nitrogen retention greater than zero.

According to the reviewed data in the NRC [4], the CP requirement for horses in average maintenance is 1.26 g/kg BW/d. Applying the 95% confidence interval, the minimum requirement is calculated to be 1.08 g/kg BW/d for horses having less activity, and the elevated maintenance requirement is estimated to be 1.44 g/kg BW/d for horses with a more active temperament. The CP requirements for maintenance in growing horses, pregnant mares, lactating mares, and exercising horses are in the same range of the requirements for mature horses at maintenance. Table 1 shows the maintenance requirements of CP in horses at various physiological states. Because nutrients required for deposition of fetal tissues, milk production, or exercise are not considered as the maintenance portion of the total requirements, the maintenance requirements of CP in mature horses are consistent in various physiological states. In addition to the maintenance requirements, requirements for growth, pregnancy, lactation, and exercise should be separately calculated and added to provide an adequate amount of CP (Table 2).

Because both body protein synthesis and breakdown are energy-consuming processes, nitrogen balance and apparent utilization of dietary protein are affected by energy balance [5]. When energy intake was deficient, horses showed weight losses even though they were fed 1.3 g/kg BW/d of CP, while a protein-restricted diet providing 0.7 g/kg BW/d of CP did not affect BW if energy intake was sufficient [6]. Therefore, adequate dietary energy must be fed to optimize dietary protein utilization.

The latest edition of the NRC [4] does not provide specific amino acid requirements, with the exception of the lysine requirements. The reported lysine requirements in mature horses at maintenance were evaluated by measuring nitrogen retention. By linear regression, zero nitrogen retention was obtained when lysine intake level was 36 mg/kg BW/d (the minimum requirement) while a broken-line analysis showed a plateau when lysine intake was above 54 mg/kg BW/d (the optimum requirement). Lysine requirements can also be expressed as 4.3% of CP requirements in horses at maintenance, growing, pregnant, and exercising states. In lactating mares, lysine requirements can be calculated by the following formula:

Lysine requirements (g/d)=kg milk yield/d×3.3 g lysine/kg milk+the maintenance requirement of lysine.

With the given lysine requirements, the NRC [4] recom mends estimating the requirements of the other amino acids based on amino acid composition in equine body tissues. For example, the lysine to threonine ratio in equine gluteal muscle is reported to be 100:62 [7], and thus threonine requirements can be estimated by this formula:

Threonine requirements=lysine requirements×0.62.

However, this approach may calculate requirements lower than the actual requirements because amino acid digestibility and metabolism occurred in the whole body may not be accounted.

## IMPORTANCE OF PROVIDING APPROPRIATE AMOUNTS OF DIETARY PROTEIN

### Low protein diets and supplementing nitrogen sources

The deficiency of either a specific indispensable amino acid or digestible protein in an animal’s diet can lead to protein deficiency symptoms. Generally, low protein intake will result in poor hair and hoof growth in horses [5], weight loss in yearlings [8], early fetal loss in pregnant horses [9], slower return to ovulation in mares [10], low protein concentrations in milk from lactating mares, and reduced milk intake and poor growth rate in foals from mares that received a protein-deficient diet [11].

Non-protein nitrogen, such as urea, has been accepted as a protein supplement in ruminant diets because rumen microbes can use urea to form bacterial proteins, which can then be digested in the abomasum and the small intestine [12]. Horses also have microbial fermentation; however, it occurs in the hindgut, which comes after the small intestine, the major site of amino acid and urea absorption [13]. There are conflicting reports in the literature as to the value of urea in replacing dietary CP in horses. In one study, a high concentration of urea fed to mature horses did not seem to be harmful [14], and in another study, the addition of urea to horse feeds resulted in increased nitrogen retention [15]. However, other studies reported that urea was primarily absorbed in the small intestine and was excreted via urine, and thus urea utilization by hindgut microbes or the host animal was marginal [13,16]. In one study, feeding a single dose of 450 g urea to 8 mature ponies (up to 136 kg of BW) resulted in the death of 7 ponies, and these ponies had increased blood urea, ammonia, α-ketoglutarate, glucose, and pyruvate concentrations [17]. Hindered α-ketoglutarate decarboxylation was suggested as the principal cause of ammonia poisoning. Therefore, the provision of urea as a nitrogen source to horses may not always be safe or efficient, so it is not recommended.

To supplement animal feeds deficient in one or more of indispensable amino acids, crystalline amino acids can be used. Historically, lysine [1,18] and threonine [1,2] have been thought to be the most limiting amino acids in typical equine diets, and they are frequently supplemented as crystalline forms in feeds to improve body protein synthesis. In previous studies, growing horses fed additional lysine showed increased growth rate [18,19], and threonine supplementation resulted in greater girth gain [1]. Also, supplementation of lysine and threonine together to mature horses improved muscle mass scores and lowered body condition scores with no decrease in BW, suggesting improved lean tissue accretion [2]. Therefore, supplementing crystalline amino acids could be an option for providing more amino acids in feeds if needed, but it does not deduct the importance of requirement evaluation.

### Consequences of overfeeding protein

Because actual requirements are unknown, one of the common strategies to ensure sufficient indispensable amino acid intake is providing a diet with high CP composition [20]. A survey study conducted on horse farms in the Chesapeake Bay Watershed region, which encompasses parts of six states in the USA (Delaware, Maryland, New York, Pennsylvania, Virginia, and West Virginia) and Washington, D.C., with 201 horses reported that the CP intake from that area was 157%± 41.8% of the NRC [4] recommendations [21]. Although practical, feeding a high-protein diet results in an elevated nitrogen content in both urine and feces of ponies [22], and the greater rates of nitrogen excretion can lead to environmental acidification and eutrophication [23]. In addition, it can cause adverse effects on animal and handler health by generating noxious gas, which irritates the eyes and the respiratory tract [24].

Excess dietary amino acids, which are not used for pro tein synthesis or other metabolism in the body, will not be stored and must be catabolized to CO_2_ and urea for excretion. Urea synthesis is an energy-consuming process. Thus, excess protein consumption would cause inefficiency in animal production. Pigs fed greater amino acids resulted in less efficient body growth associated with increased heat production (when >16.7% CP fed) and decreased energy retention (when >18.9% CP fed) [25]. During the finishing phase, feeding a high-protein diet (35.9% CP) to pigs resulted in decreased feed intake, rate of gain, and feed conversion efficiency compared to feeding the control diet (13.1% CP) [26]. In mature horses, even though the animals were fed a high-protein diet (16.6% CP), which is 160% of the CP requirement, and provided greater energy (5.5 Mcal/100 kg BW/d), they did not gain more weight than the animals fed a control diet (12.5% CP and 4.8 Mcal/100 kg BW/d) [27]. These adverse results might be due to the extra energy used to convert excess nitrogen to urea.

Moreover, greater nitrogen intake might also have nega tive effects on exercising animals. Because water is necessary for nitrogen excretion, it was found that Standardbred horses fed CP at 160% of the requirement (16.6% CP) showed increased water intake, nitrogen excretion, and urine volume [27] compared to horses received the recommended intake of CP (12.5% CP) based on the NRC [5]. Thus, overfeeding protein might be an unnecessary challenge for exercising horses as it could cause them to carry more water weight during exercise. Also, a lower blood pH resulted from feeding a high-protein diet (14.5% CP) to horses at rest and exercising compared to feeding a low-protein diet (7.5% of CP) [28]. Vigorous exercise itself could reduce blood pH by lactic acid production [29], and thus any additional decrease in blood pH caused by high level of protein consumption might increase the risk of acidosis. Because horses are an athletic species, the deterioration of exercise capacity would be a greater concern for horses compared to the other livestock species, which are primarily raised as human food sources. Therefore, feeding a high-protein diet for ensuring sufficient indispensable amino acid intake needs to be considered more carefully in horses, emphasizing the necessity of evaluating amino acid requirements.

## PROTEIN DIGESTION AND BIOAVAILABILITY IN HORSES

To optimally investigate amino acid requirements in horses, the digestive system in this species has to be well understood. Horses, an herbivore, have evolved to digest plant material. Thus, their digestive system has been developed to utilize fiber contents better compared to carnivorous or omnivorous species; microbial fermentation in the hindgut enables horses to use nutrients from plant sources. Because of the unique features of the equine digestive tract, different feed ingredients are digested with a different degree in different parts of the intestinal tract. Horses are non-ruminant hindgut fermenters, and thus they possess both mammalian enzymatic digestive capacity in the foregut (the stomach and the small intestine) and microbial fermentation in the hindgut (the cecum and the colon). Dietary protein needs to be degraded into individual amino acids or small peptides to get absorbed in the digestive tract. There are limited data regarding how much of dietary amino acids are actually absorbed and utilized for protein synthesis in horses.

### Protein degradation

Protein is digested by enzymes produced in the foregut and the pancreas, and the resulting free amino acids are absorbed in the small intestine. Hydrochloric acid secreted by the parietal cells in the stomach denatures ingested protein and activates an inactive form of the proteolytic enzyme, zymogen pepsinogen, into pepsin. Activated pepsin cleaves larger peptide chains into smaller peptides, which then enter the small intestine. Further degradation of peptides occurs in the small intestine. Compared to other parts in the equine intestinal tract, the ileum possesses the highest proteolytic activity where 23.85 μg of protein gets hydrolyzed per mg ileal content per minute [30]. In the equine ileum, trypsin, an endoprotease produced in the pancreas, hydrolyses the C-terminal of peptide bonds of amino acids. The oligopeptides broken down from trypsin activity are degraded by pancreatic carboxypeptidases, and then those constituents end up as di- or tripeptides or free amino acids by the action of the enzyme, oligopeptidase, located in the brush border of mucosal cells.

In the hindgut, microbes ferment structural carbohydrates and nutrients associated with fiber structures that are not pre-cecally digested. Thus, cell contents, including protein, which are surrounded by the structural carbohydrates of plant cells, can be finally degraded. Microflora in the hindgut ferments dietary nitrogenous components, such as gelatin, casein, peptones, amino acids, or ammonia, contributing to hindgut amino acids metabolism, but urea is barely fermented by the equine hindgut metabolism [16]. Bacterial isolates obtained from cecal contents of mature horses were cultured on a habitat-simulating medium containing urea, ammonia, peptones, or amino acids as the sole nitrogen source and only 17.9% and 20.5% of bacterial isolates were able to use urea and ammonia for the growth, respectively while 100% and 35.9% of bacteria grew in the peptone and amino acid media, respectively [16].

### Amino acid absorption

Despite the fact that 11% to 30% of total tract apparent nitrogen digestion occurs in the small intestine and 40% to 70% of it occurs in the hindgut [31,32], the majority of amino acids seem to be absorbed in the foregut, which is supported by an amino acid transporter mRNA study [33]. In the foregut, 16% to 58% of net nitrogen absorption occurs, especially in the jejunum and the ileum [31,34]. Although the nitrogen absorption in the equine small intestine has not been fully defined, nitrogen is absorbed in the small intestine mainly as amino acids in most mammals [35].

Post-ileal nitrogen uptake accounts for a significant portion of total nitrogen absorption in horses [31,32]. There is some evidence that nitrogen absorbed in the large intestine is in the ammonia form [32,36,37] but not likely in forms of amino acids [38]. Without confirmation of an *in vivo* study, some *in vitro* studies showed that L-alanine and cycloleucine were actively transported through the serosa layer, but active transport of those amino acids through the mucosa layer was not observed in the equine cecum [39,40], providing evidence that there may not be substantial uptake of amino acids from the lumen of the large intestine. On the other hand, lysine transporters across the apical membrane in the equine and swine large colon were found to have a greater capacity but less affinity than those in the jejunum, suggesting meaningful hindgut absorption of lysine [41]. However, it is still unclear whether the proteolytic enzymes exist in the hindgut to degrade microbial protein and make it available for absorption.

### Amino acid bioavailability

Amino acid bioavailability in an animal’s diet represents the proportion of absorbed dietary amino acids that can be used for protein synthesis or metabolism in the body [42]. Ileal digestibility describes amino acid bioavailability better than total tract digestibility because most of the dietary amino acid absorption occurs in the small intestine and hindgut fermentation can affect amino acid metabolism by bacterial protein production [43].

Ileal digestibility of an amino acid can be expressed in various terms: apparent, true, or standardized ileal digestibility. The differences between the values of these terms depend on how ileal endogenous losses are accounted. Ileal endogenous amino acid losses represent amino acids in ileal digesta that are not from the diet but endogenously synthesized, such as intestinal mucin protein, sloughed cells, and digestive enzymes [44]. The endogenous losses are affected by the diet that the animals consume [45,46] and divided into two components, basal and specific endogenous losses [47]. The basal losses are obligatory amino acid losses occurring regardless of which diet fed to animals. On the other hand, the specific losses are induced at different rates depending on characteristics of diet composition. Thus, total endogenous losses are also affected by feed ingredients. Feeding a highly digestible protein will result in minimal specific losses, but feeding a diet containing greater anti-nutritive factors, such as fiber, increases the specific endogenous amino acid losses [45].

Total endogenous losses can be estimated by the homoar ginine technique and the isotope tracer dilution technique [43]. The basal endogenous amino acid losses can be evaluated by measuring amino acid contents in ileal digesta when animals were fed a protein-free diet. However, in horses, feeding a protein-free diet is not a realistic method, so the basal endogenous losses may be calculated by the regression technique with nitrogen excretion in response to nitrogen intake. By subtracting the basal losses from total endogenous losses, the specific losses can be calculated.

In other non-ruminant species, protein and amino acid digestibility can be expressed in forms of apparent ileal digestibility, true ileal digestibility, and standardized ileal digestibility. While true and standardized ileal digestibilities account for endogenous losses, apparent ileal digestibility does not separate undigested dietary amino acids from total intestinal amino acid flow and thus underestimates dietary amino acid bioavailability. True ileal digestibility accounts for the contribution of both basal and specific endogenous losses, and it is the closest estimate of bioavailability of dietary amino acids. On the other hand, standardized ileal digestibility only accounts for the basal losses. Because the specific losses are not obligatory for every situation but induced depending on characteristics of feed ingredients, standardized ileal digestibility is a better estimate of bioavailability for diet formulation with various ingredients when compared to true ileal digestibility, which is inevitably affected by feed ingredients.

Because the total protein and amino acid contents present in a diet cannot be entirely digested and absorbed in animals, values of requirements in a standardized ileal digestible basis are greater than requirements expressed in an apparent ileal digestible basis. Although it would underestimate the bioavailability, protein digestibility in horses is expressed as apparent digestibility due to a lack of information on standardized equine ileal digestibility of various feed ingredients. Furthermore, individual amino acid bioavailability has not been fully studied in horses; therefore, the equine industry still relies on CP digestibility to a large extent.

### Effects of feed composition on protein digestibility

Not only is forage the major feedstuff for horses, but also horses need to consume it to maintain the normal digestive tract function. However, the components of plant cell wall, such as cellulose, hemicellulose, and lignin, are not digestible by animal enzymes, and therefore, high fiber contents in animal diets decrease protein digestibility and dietary protein utilization. In pigs, pre-cecal endogenous losses of amino acids were increased by dietary fiber [43,46], and fiber contents in feeds decreased apparent total tract protein digestibility [48] and apparent ileal amino acid digestibility [46].

In horses, a greater part of hay protein digestion occurs in the large intestine by microbes while forage nitrogen digestibility in the foregut ranges only 27% to 41% [31]. Moreover, a significant amount of nitrogen absorbed from the large intestine is believed to be in the form of ammonia, which cannot be used by the animal to synthesize indispensable amino acids [31,32,36,37]. On the other hand, the majority of dietary protein from concentrate is mainly digested and absorbed in the small intestine, presumably in the form of amino acids [49], and precaecal nitrogen digestibility of grain concentrate ranges 58% to 72% [50] in horses. Therefore, the quality of forage as well as the ratio of forage to concentrate in a diet should be considered when evaluating amino acids requirements. This issue would be more pronounced in horse diets because horse feed formulation could vary by the regional or seasonal factors and, consequently, a variety of feed ingredients are used in different situations, affecting amino acid requirements.

## THREONINE AS A LIMITING AMINO ACID IN EQUINE DIETS

Body protein synthesis occurs with a certain ratio of amino acids. A limiting amino acid is an indispensable amino acid that is provided the most below its requirement, and it hinders protein synthesis if exists. Even though the other amino acids are properly or excessively provided, protein synthesis cannot continue to occur if a limiting amino acid exists.

In a typical equine diet, consisting of grasses and cereal grains, lysine is thought to be the first limiting amino acid. Previous studies showed that supplementing lysine to common feedstuffs for horses increased growth rate in young horses [1,18,19,51]. Yearlings fed Coastal bermudagrass and a concentrate, consisting of corn, oats, and soybean meal, with 0.2% of supplemental lysine (total 42 g/d of lysine) showed increased average daily gain (0.64±0.02 kg/d) and girth gain (10.1±0.46 cm) while the control group without supplemental lysine (total 37 g/d of lysine) showed 0.57±0.02 kg/d of weight gain and 9.7±0.49 cm of girth gain [1]. Also, 0.15% of lysine supplementation (total 53 g/d of lysine) to a control diet (total 46 g/d of lysine) resulted in higher feed efficiency (15.0 kg feed/gain) while 17.9 kg feed/gain of feed efficiency was observed in the control group of yearlings [18].

Threonine has been suggested to be the second limiting amino acids in a common equine diet [1,2]. Supplementing 0.1% threonine to yearling diet, with 0.2% lysine (total 39 g/d of threonine and total 45 g/d of lysine), showed greater growth (0.67±0.02 kg/d of BW gain and 11.3±0.47 cm of girth gain) than the control group with no supplemented amino acids (total 33 g/d of threonine and total 37 g/d of lysine; 0.57±0.02 kg/d of BW gain and 9.7±0.49 cm of girth gain) [1]. However, this treatment also contained higher lysine than the control diet, and thus the result might be influenced by lysine supplementation. Compared to a treatment group receiving only additional 0.2% lysine to control diet, threonine supplementation did not change weight gain, but it increased girth gain (11.3±0.47 cm for the both threonine and lysine supplemented group and 10.1±0.46 cm for the lysine supplemented group) [1]. The increased girth gain suggested that supplementing threonine might increase body protein accretion. Also, there was a suggestion that threonine might be limiting in a weanling horses diet composed of 42:58 ratio of alfalfa hay cube to corn-based concentrate containing a recommended level of CP (9.0% CP) fed to weanlings [3]. However, a recent study in aged horses (20±1.1 years old) reported that supplementing threonine with lysine and methionine did not improve protein synthesis or nitrogen retention [52], suggesting that threonine might not be a limiting amino acid in older horses receiving a high-quality forage and a commercial concentrate.

While the NRC recommendation calculates threonine re quirements in mature horses to be 53% to 62% of lysine intake, based on muscle amino acid composition [7], previous studies showed discrepancies. When mature Thoroughbred horses were fed a 3:2 ratio of forage to concentrate, estimated threonine requirements ranged 67% to 80% of lysine intake based on the responses of plasma amino acid concentration [53]. On the other hand, when threonine requirements in Thoroughbred mares received a 4:1 ratio of forage to concentrate were investigated by the indicator amino acid oxidation method and with threonine intake levels ranged 41 to 89 mg/kg BW/d (75% to 160% of lysine intake), requirements could not be specifically defined [54]. One possibility was that the requirement fell outside the test range of threonine intake levels. However, it might suggest that another amino acid was more limiting than threonine in the experimental diet, which was a typical equine diet consisting of good quality timothy hay and grain concentrate. Further studies are still required to refine threonine requirements in horses at various physiological states.

### Threonine metabolism in animals

#### Biological role of threonine

**Figure 1:**
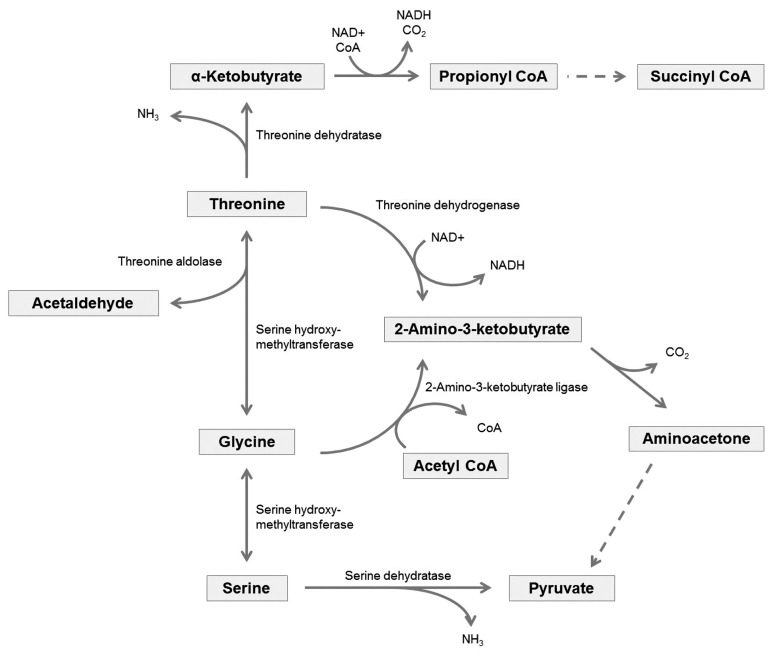
Overview of the interaction between threonine, glycine and serine degradation pathways; Adapted from Voet and Voet [61] and Wang et al [62].

Threonine, one of the indispensable amino acids in mammals, needs to be provided from feeds. In the body, it is metabolized to serine and glycine (Figure 1), which are important components of collagen, elastin, and muscle tissue [55]. Threonine has an essential role in protein synthesis. It allows proteins to fold and function correctly by glycosylation, which attaches a carbohydrate to hydroxyl or other functional groups of another molecule [56]. The hydroxyl group of the side chains on threonine is also a possible phosphorylation site, and phosphorylation on threonine is a key regulatory post-translational modification, influencing protein shapes and activity [57].

Intestinal secretory glycoprotein synthesis appears to be a major metabolic fate of dietary amino acids [58]. Intestinal mucin consists of heavily glycosylated proteins produced by epithelial cells. In particular, O-linked glycans, which are mucin type glycans, are attached to the hydroxyl oxygen of threonine and serine [59]. Therefore, threonine seems to be the significant constituent of gut mucin protein. In pigs, the contribution of amino acids from intestinal mucin to total amino acids in ileal contents was 28.0% to 33.2% threonine and 13.3% to 16.3% serine while the contribution of the other amino acids from mucin to ileal contents ranged only 1.1% to 7.1% [60].

#### Threonine degradation

Excess amounts of absorbed threonine will be metabolized by catabolic pathways producing CO_2_ and ammonia to be excreted and acetyl-CoA or pyruvate to be used in gluconeogenesis. Mammals possess several threonine catabolic pathways, and these are linked to metabolic pathways of several other amino acids (Figure 1) [61,62]. In one of the catabolic pathways, threonine is degraded into glycine, and then it will be interconverted with serine. The other threonine degradation pathway creates acetyl-CoA via an intermediate, α-ketobutyrate [63], which is also one of the intermediates of methionine catabolic pathway [64]. Due to the linkages of several metabolic pathways of amino acids, the metabolism of one amino acid could affect the metabolism of another amino acid, and vice versa [61,62].

### Factors affecting threonine requirements

#### Endogenous losses

Goblet cells line the intestinal tract and secrete mucus, termed as mucin, which is composed of 95% water and 0.5% to 5% glycoprotein [65]. In pigs fed a protein-free diet, mucin secretion was not affected by the intravenous infusion of either saline or a complete amino acid mixture [60]. This finding suggests that mucin must be secreted even if animals do not receive dietary amino acids and the animals will use amino acids from body protein breakdown to support mucin synthesis if necessary. The intestine used 52% of total dietary amino acids and 67% of dietary threonine, and 26% of dietary protein was recycled into the blood amino acid pool from mucin protein [58]. However, threonine was not found to be recycled, which means that intestinal mucin protein synthesis might be a major metabolic fate of threonine [58]. Accordingly, threonine has lower apparent digestibility (31.2% to 62.4%) compared to the other amino acids (36.0% to 92.3%) in horses [66], consistent with what has been observed in other monogastric animals [46,67]. Therefore, mucin secretion has been suggested as a substantial source of ileal endogenous threonine losses.

#### Dietary composition

Dietary fiber contents, which are also expressed as anti-nutritive factors, not only reduce nutrient digestibility but also increase endogenous protein losses by stimulating intestinal mucus secretion [45]. Because the primary role of mucin is protecting the intestinal tract from chemical digestion or physical damage, mucin secretion is inevitably increased by dietary fiber contents. Consequently, the threonine requirement is sensitive to fiber composition and quality of animal feeds [46]. In particular, hemicellulose contents from feeds resulted in reduced nitrogen retention, lower apparent ileal threonine digestibility, and greater endogenous losses of threonine in pigs [46].

#### Physiological state

While threonine requirements in horses have not been fully determined in various situations, previous research in other species suggests that various physiological states will also influence threonine requirements because the threonine needs in the body might vary for body tissue accretion, conception, or lactation. For example, the threonine requirements of neonatal piglets were 3 to 5 times greater than those of mature pigs [68,69] indicating that greater threonine intake is required for tissue accretion in younger animals. Similarly, during gestation, threonine requirements in the last third of gestation (d 81 to d 111; 12.3 g/d) were more than twice higher than requirements during early stages of gestation (d 25 to d 55; 5.0 g/d) in sows [70] due to the increase of fetal weight, fetal protein contents, and mammary protein contents [71]. Also, ideal composition of amino acids required for nursing sows depends on how much body tissue is mobilized for milk production and mammary tissue accretion [72]. When sows used more body reserves for lactating, threonine becomes more limiting [72]. Thus, the requirement of threonine in lactating animals would be higher than in animals at maintenance to prevent body protein degradation.

## METHODS USED FOR EVALUATING AMINO ACIDS REQUIREMENTS

### Calculations

#### Factorial approach

The factorial approach had been used to estimate protein and amino acids requirements in the past. This method is based on the concept that total requirements of protein can be calculated by the sum of i) the obligatory protein losses, which determines maintenance protein requirements, and ii) the protein used for synthesis of new tissues, such as growth, pregnancy, and lactation.

When animals are adapted to a protein-free diet, amino acids from the body protein are being recycled to maintain protein turnover, the flux of protein synthesis and breakdown. However, there would be a limit to the amount of amino acid recycling. In adult rats, some indispensable amino acids could be conserved when they were adapted to a low-protein diet while some amino acids from body tissues would still be degraded [73]. Thus, the ultimate limit of recycling amino acids from body protein would indicate the obligatory protein losses, and the maintenance requirement can be determined at this point. Amino acid requirements for the formation of new tissues can be estimated by measuring the rate of the tissue formation, the composition of that tissue, or the efficiency of amino acids uses for the tissue formation. Then, the requirements for maintenance and accretion of new tissues are summed into the total requirements that animals need. In the most recent NRC publication for horses [4], the factorial method is used for evaluating the requirements for growing, pregnant, and lactating horses. However, this method was developed on an assumption that the obligatory nitrogen losses are fixed in an animal which is not fully validated.

#### Amino acids composition in the animal tissue

A previous study suggested that if the indispensable amino acid profile in a diet resembles the ratio of amino acids required for structural and functional needs in the animal, the diet can be rated as having a higher biological value of protein and thus less amounts of that feed could still meet the requirements [74]. Applying this concept, amino acids requirements in horses have been estimated from body tissue composition. Because the requirement of lysine has been reported in horses (54 mg/kg BW/d for mature horses at maintenance) [4] and amino acid composition of muscle or milk can be measured, the other amino acids requirements can be calculated based on those numbers.

Although convenient, this method may underestimate requirements because it does not consider digestibility or other uses in the body. Especially, horses consume substantial amounts of forage in their diet, and nitrogen digestibility is remarkably different between forage and concentrate; estimated true prececal nitrogen digestibility of forage was 27% to 41% [31] while precaecal nitrogen digestibility of concentrate was 58% to 72% [50]. In addition, up to 60% of dietary threonine is used up in the intestinal tract in other non-ruminants [68], and threonine is considerably incorporated into intestinal mucin protein [60]. Thus, if threonine requirements were estimated based on muscle or milk amino acid composition, this estimation would not include the potentially large threonine uses in the gastrointestinal tract.

### Dose-response feeding trials

**Figure 2:**
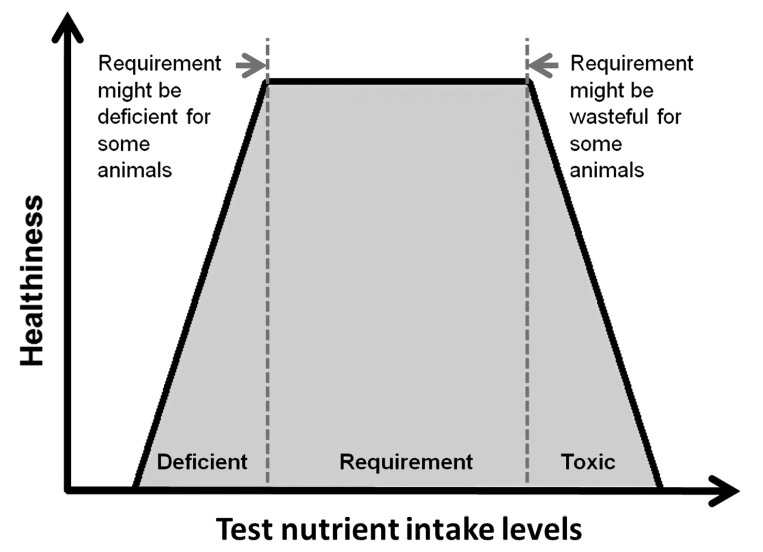
Health index curve relative to test nutrient intake levels below and above the requirement.

To measure nutrient requirements using a dose-response approach, at least 4, or ideally 6 or more, levels of test nutrient intake are required [75]. Also, the test nutrient intake levels should span both below and above the actual nutrient requirement. Along with feeding the graded levels of the test nutrient, one or more indexes of healthiness, such as growth rate and body protein retention, are measured to estimate requirements. The measured parameters would show a certain response as the test nutrient intake levels increase towards the requirement, and then a breakpoint in the response will be detected once the intake level meets the actual requirement. Until the upper limit of the requirement, providing greater amounts of the test nutrient is not harmful to animals but only wasteful, but above that limit, the excess amount of the test nutrient may result in toxicity, decreasing the indexes of health (Figure 2).

Comprehension of amino acid metabolism in animals is required to decide which outcome parameter to measure and to interpret the meaning of responses shown by the outcome parameter. Briefly, dietary amino acids will be either utilized in the body or excreted. Amino acids are mainly absorbed in the small intestine to the blood and used for protein synthesis or converted to other metabolites. If not utilized, excess amino acids will be catabolized; nitrogen from the excess will be converted to urea and carbon skeletal backbones will be either used for gluconeogenesis or oxidized to CO_2_ to exit the body via expiration.

**Figure 3:**
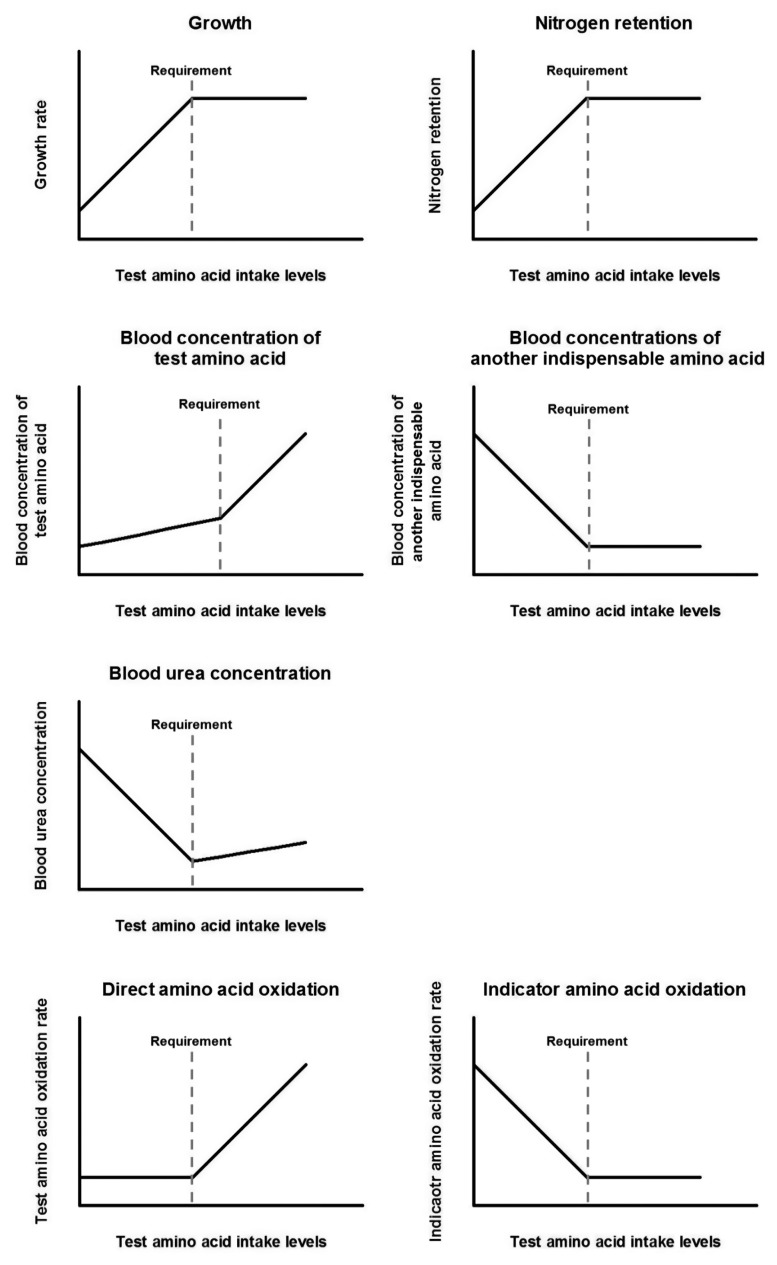
Curves of an indicator response to test nutrient intake levels below and above the requirement; Adapted from Pencharz and Ball [88].

With this biology, amino acid re quirements can be determined by various outcome parameters, such as growth rate, nitrogen balance, blood amino acid concentrations, blood urea concentrations, and amino acid oxidation rate. The responses of different indicators to increasing intake levels of a test amino acid are depicted in Figure 3. Although diverse options are available, evaluated requirements with different indicators might not be the same (Table 3) [76]. Also, measuring a certain indicator might not be appropriate for every different situation, and an estimate from a single indicator might not properly represent the actual requirement by itself. There are pros and cons corresponding to each of the various indicators.

#### Growth rate

In a previous study, the effect of dietary amino acid composition on body protein deposition was evaluated to estimate amino acid requirements for normal growth in infants [77]. This study was the first approach to estimate amino acid needs by measuring growth rate. In growing animals, measuring growth indicators can be used to determine amino acid requirements by estimating an optimal intake level of a test amino acid for maximizing growth, representing increased body protein synthesis. Growth rate will increase with greater test amino acid intake levels until the consumption reaches the actual requirement and then maintain the maximum growth rate. Measuring growth rate is one of the most non-invasive methods for studying amino acid requirements, and growth rate in horses can be measured in various forms of response variables, such as BW, height, and girth circumference.

The growth rate method of determining requirements needs a relatively high number of animals and a long experimental period. In a previous study, the effect of dietary protein levels on growth rate was evaluated with 24 foals and three different protein intake levels (8 animals/treatment) for a 12-month period [78]. In another case, while 22 foals were studied with two protein intake levels (11 animals/treatment) for a 14-month period, the statistical power for all growth parameters measured was still low (α = 0.05; power = 0.050 to 0.238) [79]. The use of this method is limited in mature animals and may be infeasible in animals with slow growth rate. Besides, it is debatable whether or not faster growth rate in growing horses indicates desirable growth.

#### Nitrogen balance

The principle of the nitrogen balance method for evaluating amino acid requirements is based on this formula:

Nitrogen retention=nitrogen intake-nitrogen excretion

The difference between the nitrogen intake and the amount excreted in feces, urine, and other forms of minor losses would represent nitrogen retention. In this method, several levels of the test amino acid are fed to animals, and feed intake, fecal excretion, and urinary excretion are measured. Then, nitrogen intake, digestibility, and excretion can be estimated, and with these values, nitrogen utilization is evaluated. The zero-balance point of nitrogen, representing nitrogen equilibrium, indicates the maintenance requirement, and the maximized positive balance represents nitrogen required for growth, conception, or milk yield.

There are some practical difficulties associated with con ducting a nitrogen balance study such as measuring accurate feed intake, collecting feces and urine as well as counting other routes of nitrogen losses [80]. The adaptation time required for achieving a new nitrogen balance responding to a nitrogen intake level of each treatment might take about 7 d in human [81]. This relatively longer time taken for adaptation can limit the application of this method. Also, this *in vivo* digestibility trial in horses has some more limitations compared to swine and ruminant studies because of high costs associated with the number of required animals and human labor in addition to difficulty of confining horses in a limited space to collect feces and urine [82]. Although the current NRC [4] protein requirements are based on the compilation of the nitrogen retention data from many independent studies, little or no studies have evaluated requirements specifically using this technique alongside graded levels of amino acid intake. Antilley et al [83] measured nitrogen balance while feeding horses with three levels of amino acid intakes. The aim of this study was to evaluate the nitrogen retention measurement as an outcome parameter for amino acid studies, but the results indicated that it was not an effective response variable for this purpose.

#### Blood amino acid concentrations

When graded levels of a limiting amino acid are fed, the blood levels of that amino acid will be minimal until the intake meets the actual requirement because the amino acid entered the blood would be mostly utilized for protein synthesis. Then, further consumption will start to increase its concentration in the blood. Although this approach has an advantage as a relatively non-invasive method, measuring blood amino acid concentration might not be an optimal method for determining amino acid requirements. In a previous study [84], plasma lysine concentrations were measured to evaluate its requirement in mature Thoroughbred horses, and the estimated lysine requirement was 72 mg/kg BW/d, which is higher than the recommended value, 54 mg/kg BW/d, in the NRC [4]. Also, threonine requirements in mature horses estimated based on blood amino acid concentration ranged 67% to 80% of lysine intake [53], while the requirements estimated based on the NRC [4] recommendation ranged 53% to 62% (applied lysine to threonine ratios in equine milk and muscle) [7]. These discrepancies might be due to the other factors affecting blood amino acid concentrations, such as time after feed intake [85] or the health of the subjects [86].

#### Blood urea concentrations

Nitrogen from excessive amino acids in the blood will be converted into ammonia and then subsequently to urea for excretion. Therefore, blood urea nitrogen concentration will increase when protein intake is excessive [87]. When graded levels of a test amino acid are fed, the pattern of plasma urea concentrations will keep decreasing until the intake level meets the actual requirement as more protein can be synthesized, and therefore, fewer amino acids will be catabolized. At the actual requirement, the plasma urea concentration will reach the minimum level, and it will increase moderately with further consumption because an excessive amount of the test amino acid will not be further utilized for the body protein synthesis and need to be degraded. However, blood urea nitrogen concentration alone does not seem to be sensitive enough as an indicator for evaluating amino acid requirements in other species [88]. Because blood urea nitrogen concentration is affected by not only body protein synthesis but also feed consumption [89] or exercise [90], this parameter only indirectly represents dietary amino acid utilization and thus needs to be reported with other types of parameter measurements to define more accurate requirements.

### Isotope methods

The recommended protein and lysine requirements in the NRC [4] were extrapolated from nitrogen balance studies. However, using isotopic tracers for evaluating amino acid requirements, a relatively new methodology, has been suggested to be more sensitive than the nitrogen balance approach. There are two main ways to apply isotope tracers to evaluate amino acid metabolism in the body: measuring direct amino acid oxidation or indicator amino acid oxidation. Infusion of amino acid tracers to the blood and oral administration of labeled amino acids are used to study protein or amino acid metabolism [91]. The principle of the isotope methods is that when excess amino acids are degraded the carbon backbones will be oxidized to CO_2_ [88]. Therefore, administration of a tracer labeled with isotopic carbon will produce labeled CO_2_ in the expiration, such as ^13^CO_2_ or ^14^CO_2_ from stable labeled isotope tracers and radioactive isotope tracers, respectively. The labeled CO_2_ production rate depends on the uses of the test amino acid in the body.

An advantage of the isotope methods is that a relatively short adaptation period between each level of test amino acid intake is needed to change the profile of the blood amino acid pool [88]. Plasma amino acid concentrations reflect changed dietary amino acid profiles in treatments within 1 day in horses [84]. In pigs, regardless of age, adaptation time (1, 2, 5, 6, 9, and 10 d) to various levels of amino acids intake (50%, 100%, and 200% of the requirement) [92] had no effect on obtaining a plateau in the isotope amino acid indicator oxidation [93]. The shorter adaptation time makes it possible to conduct studies in the narrower time frame and therefore reduces effects of the within subject variation caused as time passes, in cases of studies with growing, pregnant or lactating animals. Furthermore, the isotope methods enable the estimation of whole-body protein metabolism because the measured variable, labeled CO_2_ enrichment in exhalation, reflects the use of amino acids that would be derived within the whole body, not just dietary amino acids. On the other hand, there are still some issues associated with the isotope methods [91]. While intestinal amino acid endogenous losses and microbial amino acid synthesis might affect amino acid requirements, their contribution on the requirements cannot be separately estimated.

#### Direct amino acid oxidation method

With the direct amino acid oxidation technique for evaluating amino acid requirements, the test amino acid is administered in an isotopic form. The oxidation rate of the test amino acid will stay minimal and constant before the consumption meets the actual requirement. Then, the test amino acid oxidation rate will rise as the consumption increases above the requirement because further provision of the test amino acid will not result in more protein synthesis. There are some limitations for using this method for measuring amino acid requirements. A test amino acid to be studied should directly release its carboxyl group to the bicarbonate pool, so the labeled carbon can be excreted in breath CO_2_. Therefore, the direct amino acid oxidation method can be applied for studying metabolism of the branched chain amino acids, phenylalanine and lysine, while other amino acids, such as threonine, cannot be studied using this method [94]. In addition, designing a proper range of the test amino acid intake levels might be challenging because the test amino acid itself is the isotopic amino acid; the minimum intake level of the test amino acid depends on how much the isotopic amino acid is needed to obtain detectable labeled CO_2_ enrichment in breath [88]. While the direct amino acid oxidation technique has been used in other species including humans, it has not yet been used to study amino acid requirements in horses.

#### Indicator amino acid oxidation method

The indicator amino acid oxidation method has been refined and validated a “gold standard” technique for studying whole-body protein metabolism and amino acid requirements. In this procedure, an adaptation period to the experimental diet is followed by a constant infusion of an isotopic indicator amino acid, and sampling of breath and blood occurs throughout the infusion period. Once the test amino acid is provided at or above the actual requirement, the indicator amino acid would be used at a constant and maximum rate for body protein synthesis. Therefore, the oxidation rate of the indicator amino acid would be also consistent and minimal. The indicator amino acid should be an indispensable amino acid, and it should not be metabolically related to the test amino acid. Phenylalanine has been commonly used as an indicator amino acid because it directly releases the carboxyl group to the bicarbonate pool, so the labeled carbon can appear in exhalation. This method has been proven to be successful to use for determining amino acid requirements, identifying limiting amino acids, and estimating the metabolic availabilities of amino acids in previous studies with various species [95–97].

In horses, when this technique was applied to evaluate ly sine and threonine requirements, a breakpoint could not be identified in both cases [54,98]. Some possible reasons for the lack of a breakpoint are i) existence of another limiting amino acid in the experimental diets, ii) ranges of graded test amino acid intake levels fallen outside of the actual requirements, or iii) provision of high-quality forage. In other species, such as humans and pigs, greater knowledge of the requirements has been accumulated than in horses, and thus it enables to design basal diets that are deficient in only the limiting amino acid and to set a proper range of test amino acid intake levels. Furthermore, to formulate precise amino acid composition in experimental diets, it is inevitable to provide a significant amount of free amino acid. Horses are, however, less amenable to such diet compared to other non-ruminant species and require fiber contents in their diet to maintain gastrointestinal health. Therefore, although the indicator amino acid oxidation method shows promise for use in horses [3,99], this technique still needs additional refinement with careful experimental diet formulation.

## CONCLUSION

The research area around protein and amino acid requirements in horses requires further investigation. This is particularly true for threonine, which has been thought to be the second limiting amino acid, but where the current knowledge of its requirements is still limited in various physiological status. In this review, we summarized the importance of amino acid requirement evaluation and the information that needs to be cautiously considered prior to designing an amino acid requirement study in horses. Among the useful methods for estimating amino acid requirements, the indicator amino acid oxidation method could be applied to a wide range of subjects, and it is also thought to be a more sensitive technique because the outcome would reflect digestibility and protein turnover in the body; however, additional refinements to the methodology are necessary and caution must be taken to ensure that diets are formulated appropriately. Further research in this area would enable the industry to achieve greater productivity by economic and well-balanced feed formulation.

## Figures and Tables

**Table 1:** Maintenance crude protein requirements in horses at various physiological states1) (g/kg body weight/d)

Items	Maintenance	Growth	Pregnancy	Lactation	Exercise
	-	1.44	1.26	1.44	Same as maintenance requirement
Minimum	1.08	-	-	-	-
Average	1.26	-	-	-	-
Elevated	1.44	-	-	-	-

1) Data from NRC [4].

**Table 2:** Additional crude protein requirements in horses at various physiological states1) (g/kg BW/d)

Items	Additional crude protein requirements
Growth	(Average daily gain kg × 0.20)/E2))/0.79 g CP/d
Pregnancy	(Fetal gain kg/0.5)/0.79 g/d
Lactation	Milk production kg/d × 50 g CP/kg milk
Exercise	
Light	BW × 0.089 g CP/kg BW/d
Moderate	BW × 0.177 g CP/kg BW/d
Heavy	BW × 0.266 g CP/kg BW/d
Very heavy	BW × 0.354 g CP/kg BW/d

BW, body weight; CP, crude protein.

1) Data from NRC [4].

2) E, efficiency of use of dietary protein; 50% for horses 4 to 6 months of age, 45% for horses 7 and 8 months of age, 40% for horses 9 and 10 months of age, 35% for horses 11 months of age, and 30% for 12 months of age or older.

Total crude protein requirement can be calculated by adding the calculated values to the appropriate maintenance requirements (Table 1).

**Table 3:** Estimated indispensable amino acid in adult human using different methods1) (mg/kg body weight/d)

Items	Nitrogen Balance	Plasma amino acid concentration	Direct amino acid oxidation	Indicator amino acid oxidation
Histidine	8 to 12	-	-	-
Isoleucine	10	-	-	-
Leucine	14	30	27	-
Lysine	12	32	35	36
Methionine + cysteine	13	-	-	-
Phenylalanine + tyrosine	14	30	30	-
Threonine	7	15	15	-
Tryptophan	3.5	3	-	4.3
Valine	10	20	17	-

1) Adapted from Zello et al [76].
